# Being Concerned with Substances of Concern: A Classification of Ethical Dilemmas in Circular Product Design

**DOI:** 10.1007/s11948-026-00585-0

**Published:** 2026-02-28

**Authors:** Ida Krstulović

**Affiliations:** TU Delft, Delft, Netherlands

**Keywords:** Design ethics, Circular design, Safe and sustainable by design, Value sensitive design, Sustainability

## Abstract

In a circular economy, the use of hazardous substances forces industrial designers to face ethical dilemmas that are just as complex as those in a linear economy. A system in which resources are continuously reused rather than discarded cannot afford the reintroduction of chemicals and materials that are harmful to the environment and human health. However, eliminating or substituting a substance that causes concern is not always possible due to the design requirements or a conflict of values. This classification focuses on the types of ethical dilemmas designers are met with when trying to make their designs as safe and sustainable as possible, but are unable to maximize all of the moral values involved. In order to establish some parameters, the paper generalizes these issues and categorizes them into ethical dilemmas that appear in the production stage, those that appear in the use stage, those that appear at the end-of-use stage, and those that appear between different stages of the product’s life. By categorizing the dilemmas into stages of the product’s life that they appear in, a tool is created that helps not only designers but also toxicologists, policymakers, and other stakeholders sensitize themselves to ethical issues.

## Introduction

Alex, an industrial designer, is developing a line of hospital mattresses. She wants to minimize the use of hazardous chemicals, so-called substances of concern, and their impact on human health and the environment as much as possible. She knows fire resistance is an important property for a hospital mattress because exposure to fire is an unacceptable threat to the patients’ health and safety. However, she also knows that the most common fire-protectant compounds are toxic to humans (Iqbal et al., 2017) and are the biggest water polluters in her country, the Netherlands (Marée, 2024). Because eliminating the fire-resistant property of the product isn’t an option, Alex considers the more sustainable option of using a combination of natural textiles, wool and latex. However, she remembers the 2015 scandal surrounding Ovis 21, a farming corporation believed to be an ethical wool supplier until it was proven they partake in animal cruelty practices (Gardetti, 2017). Furthermore, she remembers the raw rubber industry has a habit of exploiting its workers, among other social issues (Dunuwila et al., 2023). Since both human and animal rights are very important to her, she hesitates to make a substitution she worries she might regret later because it conflicts with her other moral values. Being the conscientious designer she is and not wanting to regret her decisions, Alex here faces an ethical dilemma. She wants to make a decision that aligns with her personal values, as well as the values of other stakeholders. She wants to ensure human health and safety, sustainability, and justice for everyone involved. Knowing it’s impossible to maximize all of these values simultaneously, she is forced to make trade-offs and prioritize one over the other. Her challenge is to navigate all the ethical issues she faces to make her product as ethical as possible.

The circular economy model, an economic system that minimizes waste, emissions, and the extraction of raw materials by reducing, reusing, recycling, and using other strategies to keep resources in the loop (Kirchherr et al., 2017), might seem faultless at first glance. However, circular product designers are often faced with such ethical dilemmas regarding substances of concern, whether they are aware of this or not. Choosing a material, and subsequently the chemicals it’s made of, will influence every stage of a product’s life, which will have ethical implications in every cycle of the circular economy. A designer may face a choice between using a single material for their product, facilitating recycling, or using many materials to create a modular design, facilitating repair and reuse (Wan & Lin, 2022). They may have to decide whether to use an environmentally friendly material (such as aluminum) if that means that the energy, and therefore carbon footprint, used in production will be increased (Prendeville et al., 2017). Closing a material loop may incentivize overproduction, while the recovery of secondary materials may come at a cost to human health (Greer et al., 2021). Translated into moral value terms, a designer has to choose between different ways of keeping their users safe, their environment preserved, or their value chain just and fair towards everyone involved.

If a designer wants to make a morally justifiable decision about their product, she needs to develop sensitivity to ethical issues that might arise at any stage of the product’s life cycle. Before a designer can get to a solution, she needs to realize she’s not there yet, and that there even is somewhere she needs to go. What’s needed is more guidance on how to recognize and reflect on ethical issues in one’s chain of production. One established approach that can aid this process of ethical awareness is Value Sensitive Design (VSD). Developed by Batya Friedman and colleagues, VSD offers a structured way to account for human values throughout the design process through three mutually informing types of inquiry: conceptual, empirical and technical investigations (Friedman et al., 2006). VSD provides a useful foundation for recognizing and articulating the value conflicts that arise in design.

Building off of on this approach, and motivated by a recent recommendation by the European Commission (European Commission Joint Research Centre, 2022), this classification is an overview of ethical dilemmas specific to the problem of integrating the values of safety and sustainability into circular product design. It is not only about categorizing dilemmas but also serves as a tool to notice patterns in design practice by showing designers what to look out for. Its goal is to help in situations where, because of the ubiquity of ethical concerns about hazardous substances, it’s hard to see the forest for the trees. Put simply, in order not to overlook ethical issues, one needs to be aware of them in the first place.

While there is existing literature on the ethical issues that arise in material selection (e.g. Blum, 2007; Greer et al., 2021) and classifications of ethical dilemmas in various other domains, such as public health, biological warfare, or robotics (e.g. Conway, 2014; Holdstock, 2006; Kapeller et al., 2020), there is no holistic depiction of the types of ethical dilemmas that appear in circular product design. This paper contributes to that. It is not intended to be an extensive, systematic taxonomy of every possible type of ethical issue in circular design, but instead a categorization tool helpful for anticipating issues one might otherwise overlook.

This approach not only aligns with the intricacies of industrial design but also advances the discussion in other areas by providing a helpful tool that adds to risk assessment (RA) and life cycle assessment (LCA) processes, which aim to identify, evaluate, and estimate levels of risks and environmental impact in the stages of a products life, respectively. Both RA and LCA involve a certain amount of subjective and normative judgment when deciding how to prioritize potential risks (Yazdi, 2018) or impact indicators necessary for defining the goal and scope of an LCA (Rosenbaum, 2017), so an ethical framework seems fitting to show that the dilemmas appearing in SSbD are not just a matter of assessing risk or the assessment of life cycle impacts, but also a matter of ethics. These dilemmas are issues that concern what the morally right or wrong choice is, what we owe to each other and the environment we live in, or what solution would ensure justice, equality, and dignity for all human beings involved. While the more technical aspects of industrial design, like the matter of integrating the functionality of the product with its aesthetics, can be resolved through empirical analysis like market research, ethical questions can only be resolved through deep reflection and moral reasoning. An ethical perspective can aid in making normative claims about what is better or worse in a given situation and, therefore, decisions that are morally justifiable. Moreover, this framework is also designed to support policymakers and industry stakeholders, promoting a comprehensive and sustainable approach to product development. Being anchored in the principles of a circular product life cycle, it contributes a novel perspective that resonates with contemporary sustainability practices and offers actionable insights for industry professionals, providing a new impulse for ethical decision-making in industrial design.

The categories this paper offers have been developed by studying relevant examples, or case studies, through a non-systematic literature review. The databases of Scopus and Google Scholar were used, with the queries being “safe-and-sustainable-by-design”, “SSbD”, “design ethics”, and “design dilemmas”. Snowballing often gave a better idea of context, and more insight into specific examples of dilemmas in material selection. Following this, another literature research was executed solely within the scope of design journal articles. Because design literature can sometimes address ethical issues differently than how these problems are approached in ethics and philosophy, a journal-specific literature research provided an insight into design terminology, specific examples of dilemmas, and research gaps that future studies could benefit from exploring.

The paper begins by clarifying the background and key concepts necessary to understand the proposed framework. Building on this, the next section introduces Value Sensitive Design as a guiding scaffold for analyzing conflicting values. The core of the paper develops a classification of ethical dilemmas structured around the stages of the product life cycle. The paper then concludes by addressing the challenges of uncertainty and the risk of regrettable solutions, highlighting the need for continued ethical reflection.

## Background and Definitions

This research is greatly motivated by the recent developments in Safe-and-Sustainable-by-Design (SSbD) recommendations. The SSbD approach is a way to integrate the values of safety and sustainability into a chemical’s, process’s, or product’s entire life cycle and to do it in the earliest stages (Caldeira et al., 2022). It pertains to innovation, the development of new products, and the redesign of existing ones (Sudheshwar et al., 2024). Different SSbD frameworks have already been published on assessing chemicals, materials, and processes (e.g. Abbate et al., 2024; Caldeira et al., 2023; CEFIC, 2022; ChemSec, 2021; EEA, 2021), and this definition applies to all of them.

If it was always possible to maximize both the safety and sustainability of a product, as well as every other relevant moral value, there would be no problem in implementing such a framework. However, sometimes the laudable aim of being sustainable in product development comes at the price of another value, and vice versa. In many cases, this can become a value conflict, a situation in which two seemingly equally important values cannot be instantiated simultaneously by the same design (van de Poel, 2015). The struggle of dealing with conflicting values is something that’s omnipresent in the design literature as well. Designers may phrase it as dealing with trade-offs in eco-design (Prendeville et al., 2017), facing the waste-resource paradox (Greer et al., 2021), or failing to design cradle-to-cradle (Bakker et al., 2010), but what they are pointing out are ethical dilemmas in safe and sustainable product design. A value conflict becomes a moral dilemma if the values at stake represent moral obligations that cannot simultaneously be met (van de Poel, 2015, p. 112).

In the context of this paper, ‘*ethical*’ doesn’t refer to a fixed set of normative claims from any one ethical theory. I do not commit to a prescriptive theory of ethics for the purpose of categorizing these issues, because I believe that doesn’t reflect how these dilemmas are usually dealt with in practice. It is also important to note here that, while VSD allows for value pluralism, that doesn’t mean it allows for moral relativism. Value pluralism assumes multiple values can be equally important, and may be in conflict with one another, while maintaining that there are limits to cultural differences in values. This avoids the central objection to moral relativism, the concern that ‘anything goes’.

To give the discussion some normative grounding without committing to a single school of thought, this framework can be interpreted through the approach of principlism (Beauchamp & Childress, 2001), which proposes a set of mid-level ethical principles (respect for autonomy, beneficence, non-maleficence, and justice). The reasoning behind these four clusters of moral principles is that they express the underlying assumptions of the universal, common morality that binds each person no matter what their particular moral viewpoints are (Beauchamp & Childress, 2001, p. 3). Such mid-level commitments can help guide ethical decision-making while avoiding the deep disagreements that arise among different normative theories of ethics. Although often presented as an alternative to principle-based theories, *care* is another mid-level ethical commitment that can be used here. Often used as an ethical framework for design (Baas et al., 2022; Frigo et al., 2023; Hamington, 2019), care ethics places moral significance on acts of care, relationships, and the vulnerability and interdependence inherent to being human. The contextualized responsiveness to the needs of particular others that care ethics offers resonates with designers, as it helps them understand their connections and responsibilities to others beyond universal rules and abstract principles, including that of beneficence (Beauchamp & Childress, 2001, p. 375). Furthermore, like the four principles used in medical ethics, care is a normatively robust concept that can be grounded in a variety of traditions such as virtue ethics (McLaren, 2001) or Confucianism (Tao, 2000), but is equally applicable from different normative standpoints. However, since a care framework can still use a theory of justice in the public sphere (Tronto, 1993, p. 138), principlism can help ensure even those not directly related to the moral agent are treated fairly and that their rights are respected (Kuhse, 1995). Together, these mid-level ethical commitments can provide guidance without the need to adhere to strict, formulaic moral rules.

As for the term ‘*dilemma*’, it is not taken strictly to mean a situation in which one is put in the logically impossible position of choosing between two equally important moral obligations. Instead, the concept is used in a more commonsensical way – a hard choice needs to be made between multiple options that seem comparably ethical or unethical, and there is no one thing that is clearly the best choice. It is important to note that in real-life scenarios industrial designers face, it is often not even known whether conflicting moral obligations can simultaneously be met or not (van de Poel, 2017). It is also possible to imagine that a material a designer chooses will end up being the worse option in one dilemma, and the better option in another. This adds layers to the difficult moral problems I will proceed to call dilemmas.

This classification of ethical dilemmas touches specifically on the topic of *substances of concern*. A substance of concern in this context is any chemical that is (or is suspected to be) a health or environmental hazard. It can be intentionally added to the composition of the product, unintentionally generated by the product throughout its use or end-of-life, or added temporarily in the production phase but not intended to be present in the final product (Arriola et al., 2022, p. 9). Common historical examples of substances of concern are asbestos and lead. Some of the more nuanced examples of today are volatile methyl-siloxanes presumed to be emitted by silicone bakeware during use (Fromme et al., 2019), or microplastics released into the soil by biodegradable packaging (Weinstein et al., 2020).

For practical reasons, a flexible definition of the values of sustainability and safety will be given. The goal of this paper is not conceptual clarification. This classification aims to serve as a tool not only for designers but also for other industry stakeholders. Delving too deep into the analysis of the concepts of safety and sustainability could be counterproductive for that goal. On the one hand, a very narrow definition of these values may limit the tool’s versatility and applicability to different contexts and scenarios. On the other hand, safety and sustainability are complex and ever-evolving concepts, so by giving strict definitions I would risk excluding certain perspectives, as well as losing explanatory power. Therefore, while the definitions will be given, they will be left somewhat open still. In the context of industrial design, sustainability is generally conceptualized as the property of a chemical, material, or product to retain its functionality without exceeding environmental boundaries during its life cycle (Aguirre et al., 2025). Safety is often defined in the design ethics literature as the absence of an unacceptable amount of risk (Doorn & Hansson, 2015, p. 494). I allow these concepts to remain open to further interpretation, not limiting the application of this classification only to cases that align with these definitions. I believe these values can and should be protected even if they manifest themselves differently at times. Finally, it is important to note that, while this type of design highlights the values of safety and sustainability, other values also need to be taken into account. When trying to design a product that’s ethical in all stages of the value chain, values such as justice or well-being necessarily come into play. This is because the ethical dilemmas this paper is about are not limited to questions of consumer safety and environmental impact but also consider the needs of workers, the communities impacted by local extractions of raw materials, or the disproportionate distribution of environmental burdens across society. The values involved are specific to the case, and their conceptualizations specific to the actors.

## VSD as Theoretical Groundwork

This research builds on the VSD approach, first introduced by Batya Friedman in the context of software development (Friedman, 1996). The guiding idea behind VSD was that technology and technological artifacts need to be designed with values like privacy or human autonomy in mind to avoid prejudice and bias. It involves three interrelated and iterative types of investigations: conceptual investigations which aim to identify stakeholders and their respective values, empirical investigations that involve the stakeholders in the discussion through methods like interviews, surveys or experiments, and technical investigations that concern themselves with analyzing the technology or product (Davis & Nathan, 2015). In this paper, VSD is not used to generate technical interventions. Instead, its role is to provide theoretical scaffolding for a more diagnostic framework. It functions here as the groundwork for reflection and ethical awareness rather than a prescriptive design methodology. The reason VSD was chosen for the purposes of this paper is because of its focus on values. Although often used for the aim of translating abstract values into concrete technical interventions, VSD is also a methodology for identifying, articulating, and analyzing values in design processes. It is not a prescriptive framework to be used as a formula for design solutions, but a formative one that guides the design process towards value sensitivity (Hendry et al., 2021). Not unlike some other VSD methods (Friedman et al., 2017), the method used in this paper builds on VSD approach by offering another strategy for the investigation, representation and elicitation of values. One difference between the method used in this paper and the stakeholder and value mapping normally done in VSD is that this paper doesn’t focus on all relevant values, including things like economic value of efficiency, but rather only those that relate to one’s moral obligations to others – moral values. Not all stakeholder values will be normatively or morally significant, but the value conflicts that are foregrounded in this paper are exactly those in which the values correspond to moral obligations. When those moral obligations cannot simultaneously be met, one is left with a moral dilemma (van de Poel, 2015). Referring back to the principles mentioned above, these moral obligations can be described as respecting autonomy, beneficence, non-maleficence, justice and care. Using VSD as a way to make ethical tensions visible gives this paper conceptual grounding and helps frame these issues as value conflicts. As it’s already designed to involve multiple phases and perspectives (or multiple actors recognizing different values at stake), this scaffolding also makes sense with the interdisciplinary perspective this paper takes into consideration.

The latest framework developed by the European Commission’s Joint Research Centre (JRC) suggests dealing with ethical issues by means of a Social Life Cycle Assessment or S-LCA (Abbate et al., 2024). This methodology mimics environmental LCA, but with impact subcategories that focus on the social risks for different stakeholders, such as working hours, community engagement, or corruption. This is compatible with the methodologies used in VSD, which similarly suggests mapping out the relevant stakeholders and their values (Davis & Nathan, 2015). However, the JRC recommends using the reference scale (or ‘type 1’) approach for S-LCA, an approach based on pre-defined reference points determined by expectations, standards or best practices. In other words, the relevant social impacts are chosen from a predetermined set of 40. This is problematic because the problem of recognizing ethical issues cannot be fully covered by existing lists or guidelines. S-LCA deals with ethical challenges we already anticipate, but doesn’t look beyond the ‘usual suspects’. Yet, if a problem is not in an existing guideline, that doesn’t mean it doesn’t exist.

VSD should not replace S-LCA in SSbD practice, but it can inform it. VSD consists of the aforementioned three procedures: conceptual, empirical and technical investigations. However, these are not done in this order. Only ever starting from the conceptual and moving towards the technical would result in the same problem that appears with S-LCA. A designer would be presented with a list of values to look out for instead of engaging in discovery and discourse within the specific design context. This is why it’s crucial to point out the VSD phases are integrative and iterative, and the most successful way of pinpointing relevant values in a case is through empirical evidence (Le Dantec et al., 2009). The conceptual investigation phase can and should be informed by empirical and technical investigation, as a designer or other actor confronted with a difficult choice needs to be fully immersed in the specifics surrounding the issue. This is why the method used in this paper is to start with cases and reflect on what makes them so difficult.

## A Classification of Ethical Dilemmas Based on the Product’s Life Cycle

I propose the most suitable way to categorize ethical dilemmas in SSbD is to divide them according to the stage of the product’s life they appear in. This categorization serves several purposes. For one, this framework is developed in order to aid ethical reflection in the process of industrial design, but also when conducting RA and LCA. A life cycle approach seems appropriate and holistic for this purpose. Just as importantly, this type of classification makes it easy to distinguish its categories as clearly as possible. Categories based on other properties or characteristics would have more overlap, since multiple dilemmas can be intertwined. An attempt to categorize dilemmas according to the values involved, for example, would be ineffective because safety and sustainability are very closely linked values, and other values, such as justice, are often tied to them as well. A dilemma regarding sustainability is not a separate thing from a dilemma regarding safety, and vice versa. A product that is not safe often cannot be safely reused in a circular economy, making it unsustainable, and a product that produces harmful emissions to the environment through substances of concern will often present a risk to human health and safety as well (Beekman et al., 2020). This is precisely what makes the designers’ choices difficult, and what makes it troublesome to find a pattern in these issues.

To further clarify these categories, examples will be given for each, and a generalized explanation will be given, showing patterns that can equip the user of this framework with recognition cues to be used in practice. Importantly, these patterns aren’t a rigid checklist. They help the designer notice when something seems like an ethical dilemma and therefore requires more attention. Having an exhaustive list of ethical dilemmas would undercut the purpose of the paper, which is to develop sensitivity to issues beyond the anticipated ones. Instead of offering all of the possible dilemmas, I aim to present illustrative patterns or sensitizing heuristics for design that don’t close off alternatives.

The general structure of all of these types of dilemmas will be akin to the structure of any moral dilemma: the agent will have a moral obligation to do each of two or more actions, but will not be able to do both or all of the actions simultaneously. The key characteristic of a moral dilemma is that the agent seems condemned to moral failure, as any alternative seems to sacrifice at least one moral obligation. Because the context is SSbD, the dilemmas in this paper will also necessarily involve preserving the values of safety and sustainability as the moral reasons or normative motivation for action.

### Dilemmas that Appear in the Production Stage

Understood broadly, production-related dilemmas are not limited only to manufacturing itself, but also the extraction of raw materials, material production, prototyping, and testing. The way they can be recognized is by acknowledging that a design issue that appears at this stage is actually a situation in which moral principles conflict. For example, if a designer is aiming for sustainability in her design, she probably believes it would be immoral for the product to actively harm the environment and human health through pollution. However, this is easier said than done. The example of choosing alternative materials for a care mattress used in the introduction is one such dilemma. The designer believes she should look into natural, sustainable material options for the product she is working on, but in doing so she risks sacrificing other moral values important to her, such as workers’ rights or animal welfare. Since no option is clearly morally better or worse, she is presented with an ethical dilemma.

Another example is the use of recycled plastics when the sustainability goal is to use the least amount of virgin (non-recycled) plastic possible. Since recycled plastic is of lower quality, recycled products will be thicker, heavier, use more of the material overall, and require more energy to be produced (OECD, 2021). The question is whether these downsides outweigh the benefits of using less virgin plastic. This dilemma might compel a designer to choose a material other than plastic altogether. Paper and cardboard, for example, are often presented in the market as a desirable alternative to plastic. However, these materials are still problematic in the production stage because resourcing them from the environment can destroy ecosystems, and manufacturing paper packaging uses significantly more energy (Deshwal et al., 2019). All of these options for packaging materials jeopardize sustainability, and force designers to evaluate which disadvantage to favor over the other – to judge what they value more and why.

### Dilemmas that Appear in the Use Stage

This kind of dilemma appears when one wants to create a product that doesn’t violate any of the aforementioned moral principles in this stage of its life. Again understood broadly, the issues that arise during the use stage are connected to the distribution of the product, the dangers the users are exposed to while using and maintaining the product, or the pollution of the environment while the product is in use.

A type of products that fall into this category are products with flame, water, and oil-resistant properties. Chemicals used as flame retardants, as well as hydrophobic and oleophobic compounds, are toxic to humans and wildlife and pollute the air and water. Some of these are per- and poly-fluoroalkyl substances (Baker & Knappe, 2022), halogenated and novel brominated flame retardants and perfluorinated compounds (Wang et al., 2017). Used in textiles, furniture manufacture, and plastics, these substances aim to protect the user from fire, water, or oil-related accidents while simultaneously making the product more durable. For lack of a better alternative, a designer has to choose whether to omit this toxic substance from their product entirely, thereby diminishing its safety and sustainability in another way. This illustrates the dilemma of choosing between protection from fire hazards and the risks of exposure to toxic chemicals, two conditions which are both important for the value of safety, but that cannot be achieved simultaneously in this specific case. A diligent designer might be faced with the impossible mission of choosing which danger to expose the user to.

### Dilemmas that Appear at the End-of-Use Stage

In the circular production chain, the products aim to be cradle-to-cradle type objects that are recyclable, biodegradable, or reusable in other ways, meaning there is no ‘end-of-life’ stage (Bakker et al., 2010). However, reintroducing a material back into the value chain comes with its own challenges, and this is only made more complicated by situations in which a product may be discontinued, but the materials and substances it consists of are reintroduced into the circular production cycle. End-of-use dilemmas therefore concern the fate of a material after its primary use. They typically emerge when products designed to be circular either fail to be circular in practice or introduce new risks, like accumulation of substances of concern or ecosystem disruption, downstream. Designers can recognize this type of dilemma whenever the intended circularity route (recyclability, reuse, recovery, etc.) cannot be achieved without sacrificing a different moral obligation.

An example is choosing a material for food packaging and delivery. Think of pizza boxes. Given that they are disposable items, a recyclable material such as cardboard seems like an acceptable option in a circular economy. However, the contaminants that stem from the food products themselves, such as oil, make the reuse of the boxes impossible and recycling improbable. This is a challenge that affects many other complex feedstocks as well, defeating the purpose of using renewable materials in the first place (Lee et al., 2023). Biodegradable polymers might then seem like a desirable alternative for food packaging. However, biodegradable packaging is less durable than plastic (Mangaraj et al., 2019) and is not recyclable like paper. In fact, not only is this material not recyclable, and therefore not circular, but it has been shown to release microplastic particles as it disintegrates (Weinstein et al., 2020). It is not yet known whether these particles pose the same threat as microplastics from conventional plastic materials, but this is hardly a desirable side effect, especially for a product that comes into direct contact with food. The dilemma in this case is having to choose between two bio-based materials that are both, in their separate ways, less sustainable than they seem at first glance.

Another interesting case are natural substances used in cosmetics. Though these substances are often used as “environmentally friendly” alternatives to artificial ones, some of them can be toxic nonetheless. Phytoestrogens, for example, are a natural ingredient claimed to be beneficial to human health but are toxic to the environment (Bucheli et al., 2018). This makes skin care cosmetics containing them safe and desirable in the use stage but damaging to aquatic wildlife exposed to them through wastewater. Terpenes and terpenoids are also chemicals found in nature, commonly used as insect repellents, that are known to be toxic to wildlife and humans (Bucheli et al., 2018). Considering all this, some natural substances might not be as safe and circular as expected. Even though they come from nature, at the end of their life, they are introduced to an eco-system they do not belong to and cause harm to it. If these substances are already used as an environmentally friendly alternative to another but are proven to be bad for the environment as well, what can a designer morally justify using in their product?

Permanently disposing of a product or material that is suspected to contain substances of concern might solve some safety concerns, but is not the most sustainable option. In the Netherlands, for example, asbestos needs to be wrapped in double plastic packaging before being disposed of in controlled landfills (Le Blansch et al., 2018). While we cannot keep reusing asbestos for the sake of the environment, disposing of it this way is not a sustainable practice in the long term. Even if a natural substance, like the aforementioned phytoestrogens, is phased out until it’s eliminated entirely, it still has to go somewhere. If this isn’t into the wastewater it’s contaminating, it will be into the landfills. Neither of these options is very sustainable, making this yet another dilemma designers should be wary of early on – the dilemma between permanent disposal and reintroducing a substance of concern into the cycle.

### Dilemmas Between Different Life Stages

Perhaps most often, dilemmas appear when choosing between materials that create ethical issues in different stages of the product’s life cycle. Cross stage-dilemmas arise when improvements at one life-cycle stage (e.g. recyclability at end-of-use) create significant drawbacks at another (e.g. energy intensity in production). These can be difficult to detect as they require designers to compare different, often incommensurable, types of burdens across life-cycle stages. They can be recognized by looking at the impacts an alternative that is better in one phase has in its other phases. If the alternative performs significantly worse in a different phase, chances are it’s an ethical dilemma.

A designer may have to choose, for instance, whether to prioritize using materials that are resourced and produced ethically or those that are more easily reintroduced into the environment by cradle-to-cradle standards. An example is the dilemma between using recycled plastics and aluminum. Aluminum, which is more (easily) recyclable than plastic, seems like a good alternative to it. However, this material requires an added process during production, polishing, increasing overall energy use. Casting aluminum is also expensive, so it’s often done in eastern Asia, where the process is significantly cheaper. Outsourcing the service increases transport emissions, burdens Asian countries with the environmental consequences of the process, and exploits cheap labor in a foreign country (Prendeville et al., 2017).

This is a dilemma that, on one hand, considers the end-of-use stage of sustainable plastics and the harsh reality that only 9% of plastic waste is recycled globally (OECD, 2022). On the other hand, the easily recyclable aluminum is problematic in the production stage. From a sustainability perspective, aluminum smelting, casting, and polishing is extremely energy-intensive, harming the environment (Garsous et al., 2023). From a justice perspective, the offshore outsourcing of services is often associated with human rights risks, such as forced labor in the major aluminum companies in the Chinese region of Xinjiang (Horizon & Advisory, 2022).

Dilemmas between life cycle stages are especially easy to overlook, particularly for less experienced designers. It is less intuitive to compare the negative impact products may have in different stages of their life cycle than to compare how two products perform in the same stage, judged against the same criteria. Of course, this only makes it more important to be aware of the existence of this type of dilemma as early in the design process as possible.

### On Using this Classification as a Tool

Table 1 offers a structured view of the process for identifying and reflecting on value conflicts throughout the product life cycle. Using the care mattress example once again, the table illustrates the process of translating design problems into ethical dilemmas in each stage of the product’s life cycle. This is by no means an exhaustive list of ethical dilemmas one might encounter in the design of this product; it is solely an example.

**Table 1:** Example of the categorization of ethical dilemmas on the care mattress case

Design phase	Design problem	Moral values at stake	Ethical dilemma
Production	Using recycled instead of virgin plastics uses more materials and energy.	Sustainability	More virgin plastic should not be produced. More material and energy should not be wasted on making plastic recyclable, especially if it is likely it will not be recycled in practice.
	Use of alternative, natural materials (latex, wool) may negatively impact humans and animals.	Sustainability, justice, animal welfare	The product should be made using organic materials as much as possible, but workers’ rights and animal welfare should not be jeopardized.
Use	Flame retardants and antimicrobial coatings may contain hazardous substances.	Safety	The product should protect the user from fire and disease/infection, but shouldn’t contain toxic or otherwise hazardous substances.
End-of-use	Material recovery requires workers to handle medical waste.	Safety, sustainability	Materials should be recovered as much as possible to ensure circularity, but worker safety should not be risked when handling medical waste.
	Durability achieved through using glues and/or additional materials hinders disassembly or other material recovery routes.	Sustainability	Product should last as long as possible, but shouldn’t produce unnecessary waste or hinder recyclability, disassembly, or other material recovery routes.
Cross-stage	Addition of materials that provide the user comfort or hygiene hinders recyclability or other material recovery routes.	Sustainability, human well-being	The user should be safe and comfortable using the product, but the product shouldn’t include additional materials that hinder recyclability, disassembly, or other material recovery routes.
	Using less material negatively impacts durability and shortens life cycle.	Sustainability	The product should be made with as little material as possible to minimize waste and energy used, but should at the same time be made as durable as possible to lengthen its life cycle.

**Fig. 1:**
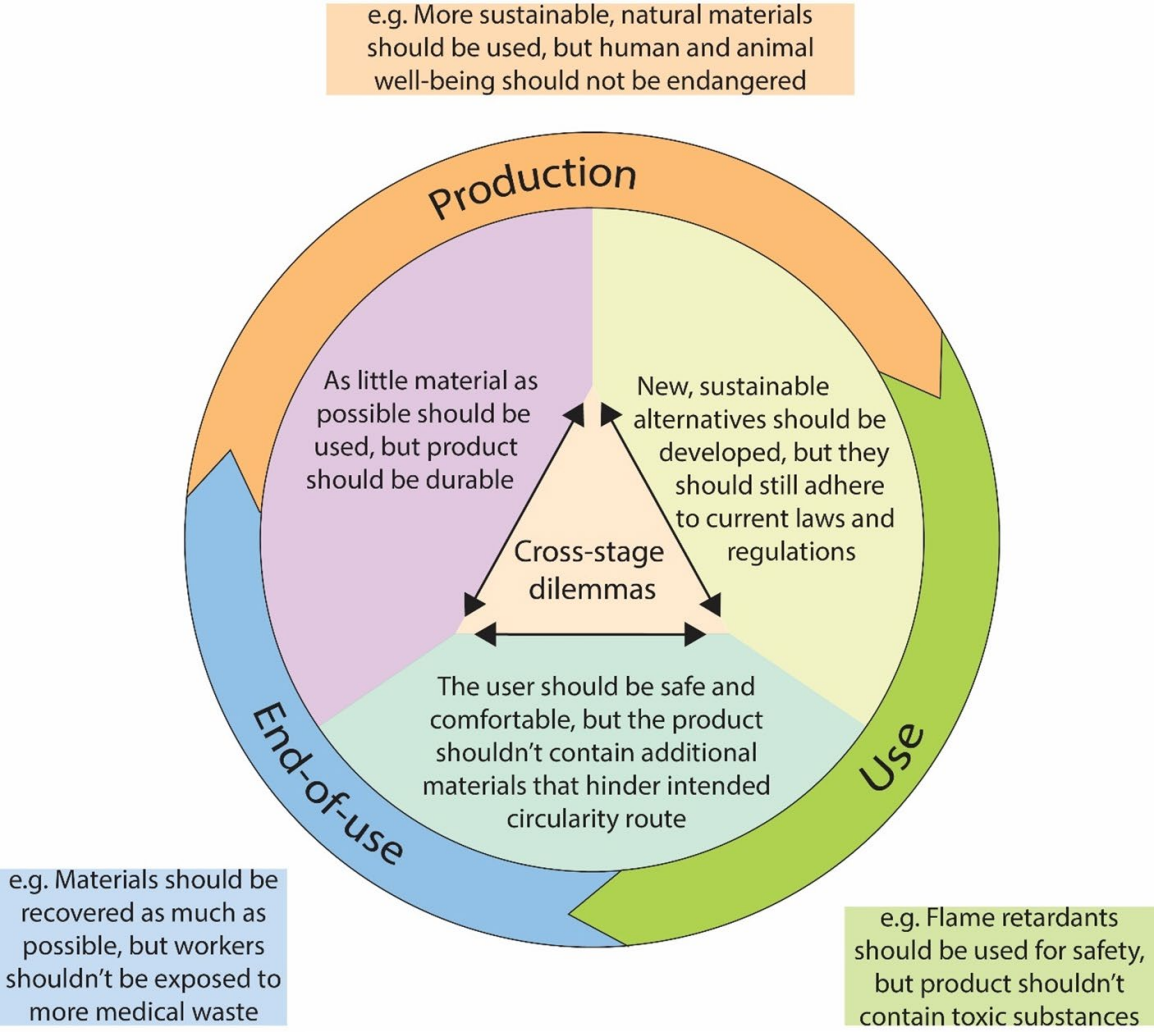
The care mattress case presented in a way that visually represents the circular economy model

Another way to illustrate this, one that will be more intuitive to those already working with the concept of circularity, would be the circular model shown in Fig. 1. This way of visualizing ethical dilemmas is especially useful in pointing out cross-stage conflicts.

This translation of design problems into ethical dilemmas at every stage of the product’s life cycle, as well as the visualization of them using the familiar cyclical shape, gives the designer a clear overview of all the ethical dilemmas they encounter working on a project. The categorization helps them make sense of these seemingly unrelated dilemmas, and making the values explicit helps them understand *why* these are dilemmas in the first place – what it is that is at stake. These examples only use one product to illustrate how this framework can make ethical dilemmas more explicit and more systematic in the design process. The same template can, however, be used to categorize any design problems that relate to moral concerns.

Before this paper is concluded, it’s worthwhile to mention the problem of uncertainties that often surround design dilemmas. Though not a category of dilemmas in itself, but more of a modifier, it is a crucial aspect of design that designers cannot always predict every impact their product will have, requiring them to be even more vigilant and sensitive to ethical issues.

## Uncertainties and Regrettable Substitutions

Dealing with uncertainties can appear in any of the aforementioned stages of a product’s life. One major element of uncertainty is data availability. The lack of available data on substances of concern is a significant issue for any SSbD framework, as pointed out in the European Commission’s Joint Research Centre technical report (Caldeira et al., 2022). Ideally, the complete compositions of products and the materials used in them would be publicly shared so an assessment can be made. However, since that often requires confidential industry information, producers often refrain from transparently giving out data (Beekman et al., 2020).

In this context, a particularly relevant type of uncertainties are moral uncertainties, situations in which stakeholders are uncertain as to how to morally interpret a situation, which values to apply, and how (Nickel et al., 2022). Even with the best intentions, designers do not have the power to predict all the possible negative consequences of choosing a specific material for production. Often, the ethical issues that may appear at a later stage of the product’s life are simply beyond a designer’s grasp (Albrechtslund, 2007). And even with all the facts, moral uncertainty might still make a designer unsure on what the right thing to do is. Moral uncertainties are also precisely why this paper tries to elucidate the values involved in making the decisions – although epistemic uncertainties can be diminished by simply gaining more information on the subject, moral uncertainties require one to elucidate the values involved, reflect on how they are conceptualized, and what other values they conflict with.

It’s important to know when to take the risk with uncertainties because of the possibility of regrettable substitutions. A regrettable substitution can be defined as the replacement of a substance that’s known to be hazardous with another substance that is just as hazardous or even more hazardous than its predecessor (Maertens et al., 2021; Ujaczki et al., 2022). Most of the time, this situation happens because of problems with data availability and quality, and the replacement chemical’s hazardous properties become apparent too late. An example of a regrettable chemical substitution in a mass-produced item is bisphenol A (BPA) in water bottles. Due to concern about its possible adverse effects on human health, this chemical was replaced by other types of bisphenols, which in turn spawned the same concerns (Rochester et al., 2015). Another well-known example is the replacement of lead in gasoline with methyl tert-butyl ether (MBTE). Though not proven to have adverse health effects in humans, MBTE is toxic to aquatic life (Tickner et al., 2019). The hazard problem is not solved, only relocated.

To deal with uncertainties regarding substitutions, designers should, whenever possible, eliminate substances of concern in ways that don’t require substitution at all. Ujaczki and colleagues (Ujaczki et al., 2022), when talking about eliminating SoC in pharmaceutics, give the following alternatives: omission from an otherwise unchanged product, termination of use of the whole product or process, gradual withdrawal of the substance by allowing the existing products to be used until the end of their life (but not producing any more), and a complete change in technology (making the product or the process of manufacturing obsolete). However, under uncertainty, these options are rarely possible, and will often introduce wholly new problems. In many cases, substitutions are the only option. This is where the true ethical dilemma lies: Is it morally better to use a substance with a high-risk level (but a lower uncertainty level) or one with a high uncertainty level and, therefore, an unknown risk level? Though risk is undesirable, a known outcome can be assessed and managed, unlike with uncertain prospects. Studies also show that the prioritization of chemicals in environmental assessments succumbs to the ‘Matthew effect’, meaning that research on hazardous chemicals focuses predominantly on substances that are known to be hazardous, instead of those that still need to be assessed for risk (Anna et al., 2016; Grandjean et al., 2011). This makes sense since many chemicals pose no threat to humans or the environment. Researching every single chemical in industrial production in order to find the pollutants is not realistic. Still, choices need to be made. For now, this remains a dilemma.

## Conclusion

Do you recall Alex, the designer I introduced at the beginning, who is grappling with the ethical dilemmas of designing care mattresses? Thanks to the classification given in this paper, Alex now has a clear understanding of the types of dilemmas she could encounter at each stage of the product’s lifecycle. At the production stage, she must choose whether to risk the human and animal welfare implications of choosing wool and latex or resign herself to the less sustainable option of using synthetic materials. She understands she is torn because her moral aim to be sustainable may come at the cost of labor exploitation or animal cruelty, both of which she is firmly against. In the use stage, she must choose whether it’s more important to keep the user safe from fire or from exposure to a toxic flame retardant. She understands this dilemma is between two specifications of the value of safety that she deems equally important because she empathizes with the user, and doesn’t want to endanger their health and safety. Translating these design requirements into ethical dilemmas helps her make better-informed, more carefully considered decisions that better align with her values and the values of other stakeholders – decisions she can defend and that she is less likely to regret in the future. It helps her reflect on what the right thing for her to do is and why, instead of just measuring impacts using quantitative methods. Unfortunately, there is no magical formula for ethics that can tell designers what the morally right thing to do is in any given situation. However, an ethical lens categorized by something they’re familiar with, the circular economy cycle, can help them notice where the challenges lie and reveal the values that come into conflict.

For designers, considering the potential ethical dilemmas is a step that fills the gap between meeting the basic design requirements and finalizing a detailed design. The design requirements for a product will undoubtedly have non-negotiable criteria regarding material selection, which limits the designer’s options. Nevertheless, before settling on a final choice for the materials, it’s essential to reflect on the moral issues that may arise, balancing conceptual investigations with the empirical and technical. What ethical concerns arise during the production, use, or end-of-use phase? Why and for whom are they concerns (that is to say, what and who are the relevant values and stakeholders)? Are there dilemmas that span across different stages of the life cycle? How much uncertainty is acceptable? And finally, what is the morally best thing to do?

Uncertainties and data gaps should not be neglected but investigated and monitored. This is where RA and LCA come in, analyzing the safety and sustainability implications of alternatives we don’t yet know much about. Some decisions will still need to be made under a lot of uncertainty, but the fewer and farther between those decisions are, the better. For policymakers, this paper demonstrates the complexity of the ethical decisions designers must make. Designers don’t always have a lot of influence over the entire supply chain, nor do they always have the final say in the matter of material selection. Whether a substance is a resource or waste often depends not on its potential but on laws, regulations, or market rules that dictate its use (Greer et al., 2021). Decision-makers should, therefore, also be aware of the issues mentioned in this paper in order for real changes to be made in the industry. Policymakers can play a crucial role in assisting designers like Alex by implementing various supportive measures. For instance, implementing incentive programs, like tax credits and subsidies, that encourage manufacturers to choose sustainable materials. Also, by establishing clear guidelines and standards for sustainable materials and design practices, policymakers can provide a roadmap for designers to follow, reducing ambiguity and simplifying ethical decision-making processes. In conclusion, collaboration is needed between all actors in the value chain to navigate these dilemmas successfully.

Future research could investigate how this classification could be applied to other fields that deal with ethical dilemmas regarding safety and sustainability, such as the textile industry, architecture, or civil engineering. Such research could contribute to the development of tools that help designers and engineers make difficult decisions in a more informed and mindful way. Another way to build upon this research is to apply existing strategies for managing ethical dilemmas in design and engineering. Van de Poel, for example, offers a number of ways to deal with value conflicts that are compatible with VSD, even when those values are incommensurable, like the (re)specification of values, setting thresholds and finding new alternatives through innovation (van de Poel, 2015). Value hierarchies are also useful for translating values into design requirements (van de Poel, 2013). It is worthwhile exploring whether these strategies are appropriate for a circular economy and conditions of high uncertainty, or whether new strategies need to be developed. This paper has illustrated what types of ethical dilemmas may appear in circular design, and why it’s important to be aware of them ahead of time. The next step is to look at how these issues can be dealt with, and how making these difficult decisions can be made easier for designers in the future.

## Data Availability

Not applicable.
